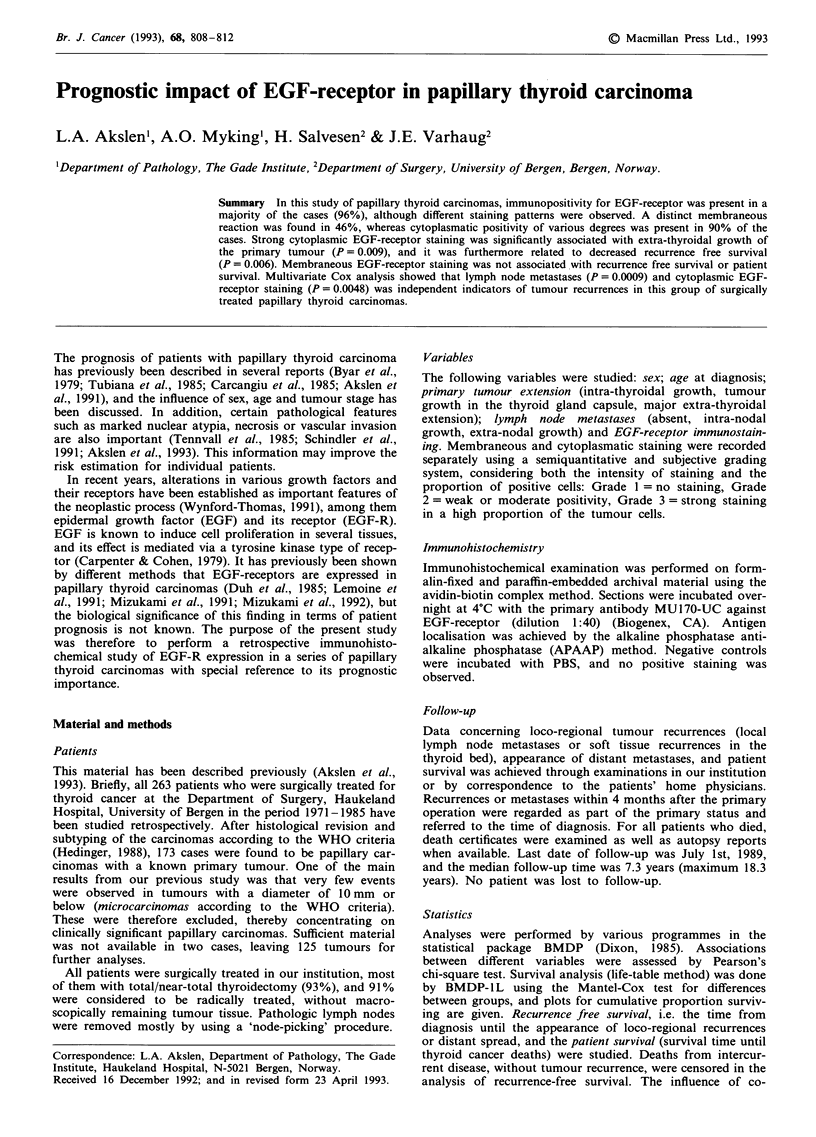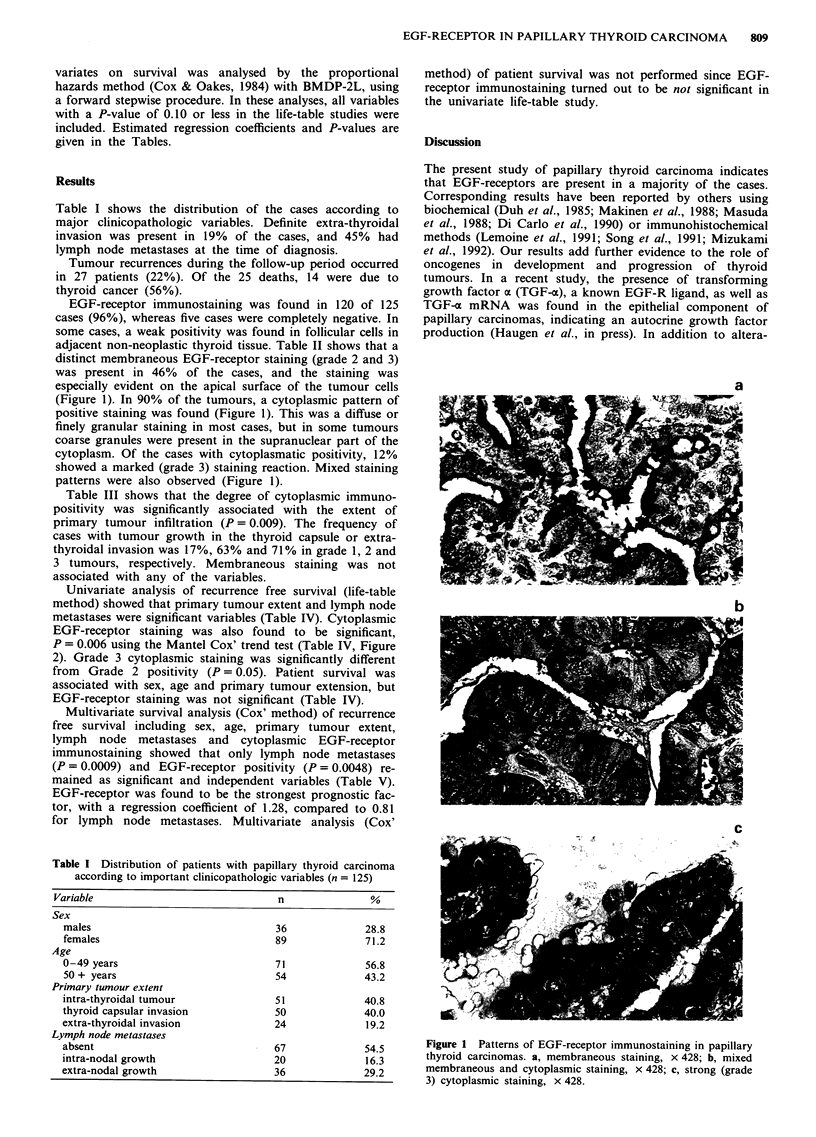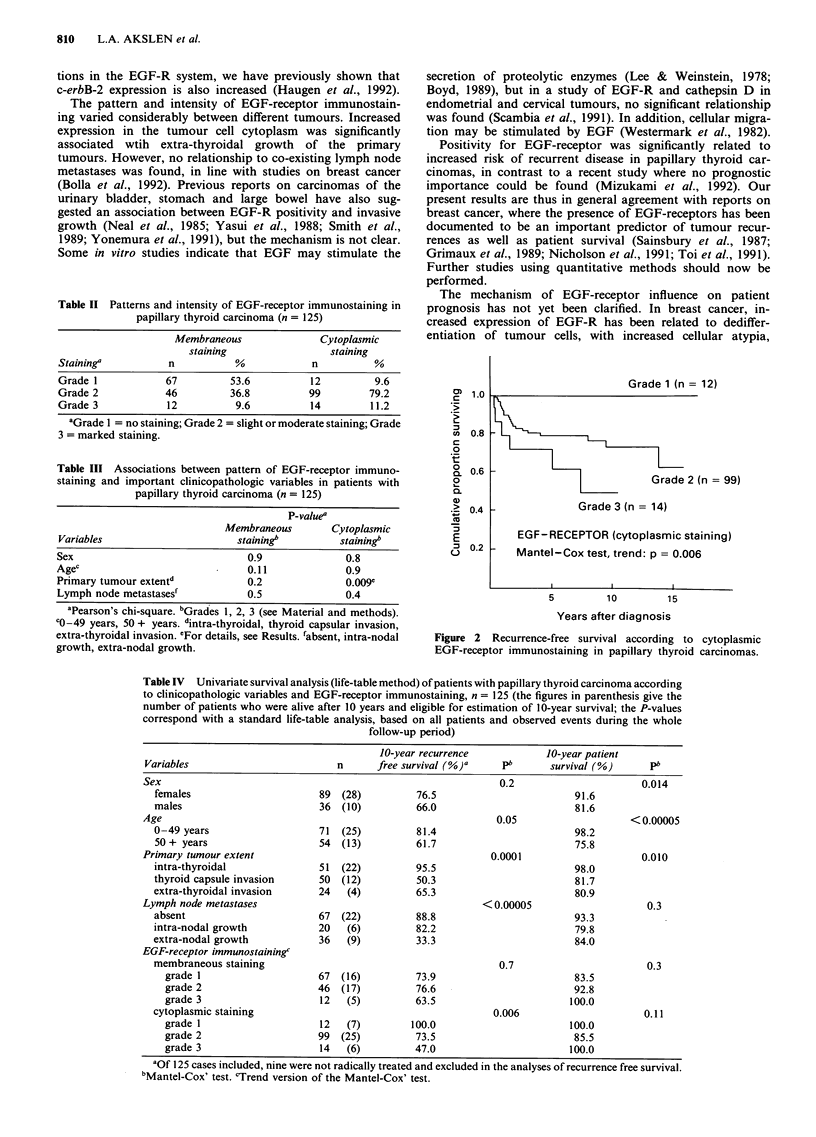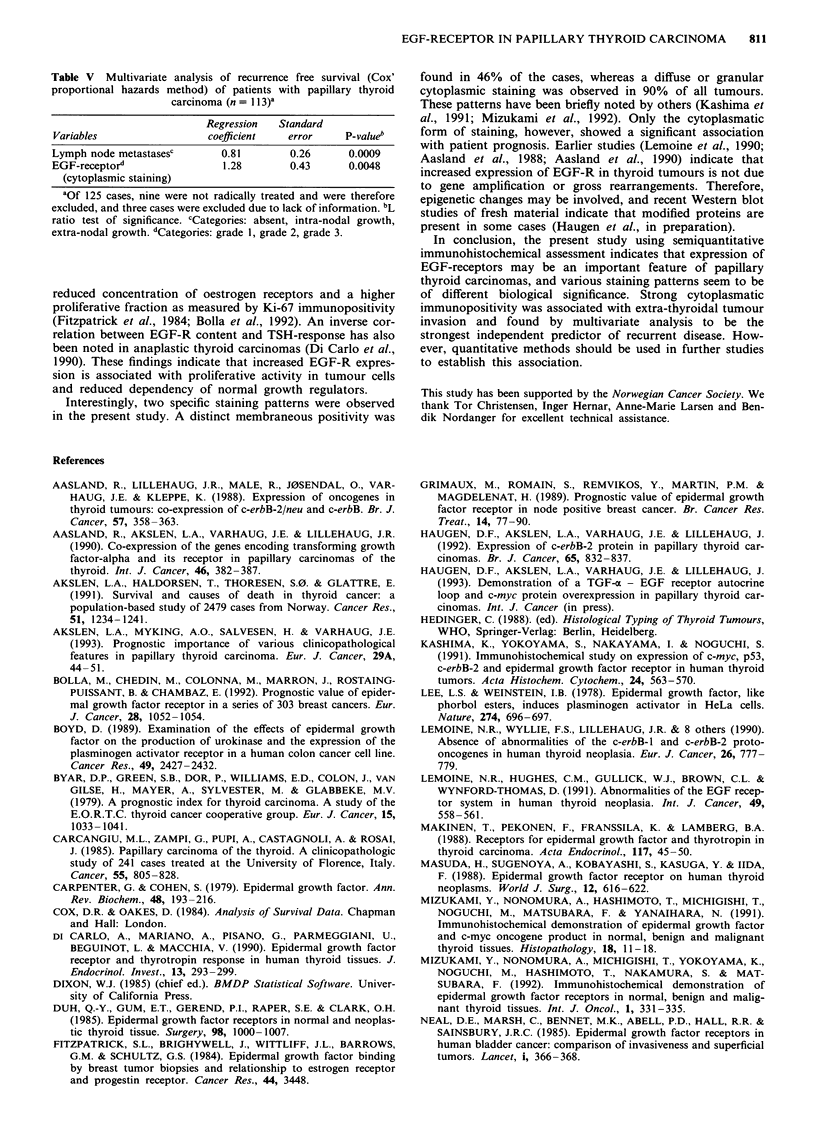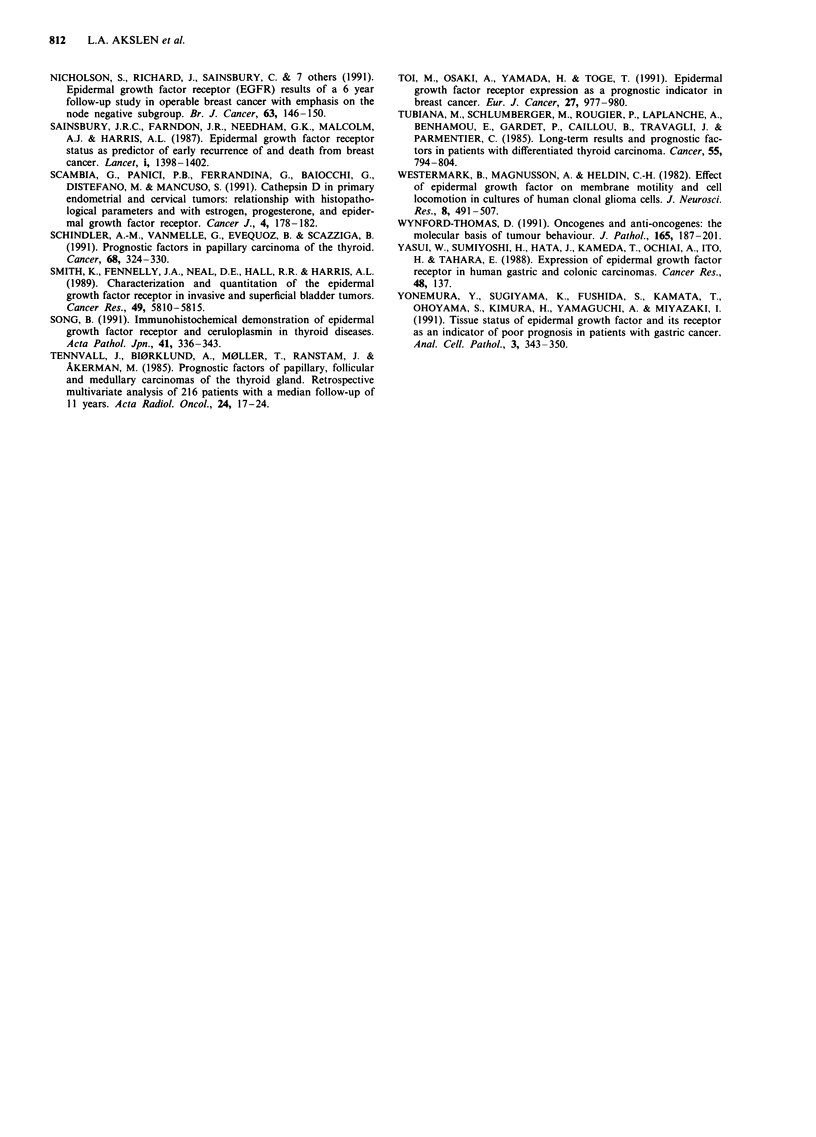# Prognostic impact of EGF-receptor in papillary thyroid carcinoma.

**DOI:** 10.1038/bjc.1993.432

**Published:** 1993-10

**Authors:** L. A. Akslen, A. O. Myking, H. Salvesen, J. E. Varhaug

**Affiliations:** Department of Pathology, Gade Institute, Bergen, Norway.

## Abstract

**Images:**


*Br. J. Cancer* (1993), **68**, 808–812 © Macmillan Press Ltd., 1993

### Prognostic impact of EGF-receptor in papillary thyroid carcinoma

L.A. Akslen[1], A.O. Myking[1], H. Salvesen[2] & J.E. Varhaug[2]

[1]*Department of Pathology, The Gade Institute,* [2]*Department of Surgery, University of Bergen, Bergen, Norway.*

**Summary** In this study of papillary thyroid carcinomas, immunopositivity for EGF-receptor was present in a majority of the cases (96%), although different staining patterns were observed. A distinct membraneous reaction was found in 46%, whereas cytoplasmatic positivity of various degrees was present in 90% of the cases. Strong cytoplasmic EGF-receptor staining was significantly associated with extra-thyroidal growth of the primary tumour ($P = 0.009$), and it was furthermore related to decreased recurrence free survival ($P = 0.006$). Membraneous EGF-receptor staining was not associated with recurrence free survival or patient survival. Multivariate Cox analysis showed that lymph node metastases ($P = 0.0009$) and cytoplasmic EGF-receptor staining ($P = 0.0048$) was independent indicators of tumour recurrences in this group of surgically treated papillary thyroid carcinomas.

The prognosis of patients with papillary thyroid carcinoma has previously been described in several reports (Byar *et al.*, 1979; Tubiana *et al.*, 1985; Carcangiu *et al.*, 1985; Akslen *et al.*, 1991), and the influence of sex, age and tumour stage has been discussed. In addition, certain pathological features such as marked nuclear atypia, necrosis or vascular invasion are also important (Tennvall *et al.*, 1985; Schindler *et al.*, 1991; Akslen *et al.*, 1993). This information may improve the risk estimation for individual patients.

In recent years, alterations in various growth factors and their receptors have been established as important features of the neoplastic process (Wynford-Thomas, 1991), among them epidermal growth factor (EGF) and its receptor (EGF-R). EGF is known to induce cell proliferation in several tissues, and its effect is mediated via a tyrosine kinase type of receptor (Carpenter & Cohen, 1979). It has previously been shown by different methods that EGF-receptors are expressed in papillary thyroid carcinomas (Duh *et al.*, 1985; Lemoine *et al.*, 1991; Mizukami *et al.*, 1991; Mizukami *et al.*, 1992), but the biological significance of this finding in terms of patient prognosis is not known. The purpose of the present study was therefore to perform a retrospective immunohistochemical study of EGF-R expression in a series of papillary thyroid carcinomas with special reference to its prognostic importance.

### Material and methods

### *Patients*

This material has been described previously (Akslen *et al.*, 1993). Briefly, all 263 patients who were surgically treated for thyroid cancer at the Department of Surgery, Haukeland Hospital, University of Bergen in the period 1971–1985 have been studied retrospectively. After histological revision and subtyping of the carcinomas according to the WHO criteria (Hedinger, 1988), 173 cases were found to be papillary carcinomas with a known primary tumour. One of the main results from our previous study was that very few events were observed in tumours with a diameter of 10 mm or below (*microcarcinomas* according to the WHO criteria). These were therefore excluded, thereby concentrating on clinically significant papillary carcinomas. Sufficient material was not available in two cases, leaving 125 tumours for further analyses.

All patients were surgically treated in our institution, most of them with total/near-total thyroidectomy (93%), and 91% were considered to be radically treated, without macroscopically remaining tumour tissue. Pathologic lymph nodes were removed mostly by using a 'node-picking' procedure.

Correspondence: L.A. Akslen, Department of Pathology, The Gade Institute, Haukeland Hospital, N-5021 Bergen, Norway.
Received 16 December 1992; and in revised form 23 April 1993.

### *Variables*

The following variables were studied: *sex*; *age* at diagnosis; *primary tumour extension* (intra-thyroidal growth, tumour growth in the thyroid gland capsule, major extra-thyroidal extension); *lymph node metastases* (absent, intra-nodal growth, extra-nodal growth) and *EGF-receptor immunostaining*. Membraneous and cytoplasmatic staining were recorded separately using a semiquantitative and subjective grading system, considering both the intensity of staining and the proportion of positive cells: Grade 1 = no staining, Grade 2 = weak or moderate positivity, Grade 3 = strong staining in a high proportion of the tumour cells.

### *Immunohistochemistry*

Immunohistochemical examination was performed on formalin-fixed and paraffin-embedded archival material using the avidin-biotin complex method. Sections were incubated overnight at 4°C with the primary antibody MU170-UC against EGF-receptor (dilution 1:40) (Biogenex, CA). Antigen localisation was achieved by the alkaline phosphatase anti-alkaline phosphatase (APAAP) method. Negative controls were incubated with PBS, and no positive staining was observed.

### *Follow-up*

Data concerning loco-regional tumour recurrences (local lymph node metastases or soft tissue recurrences in the thyroid bed), appearance of distant metastases, and patient survival was achieved through examinations in our institution or by correspondence to the patients' home physicians. Recurrences or metastases within 4 months after the primary operation were regarded as part of the primary status and referred to the time of diagnosis. For all patients who died, death certificates were examined as well as autopsy reports when available. Last date of follow-up was July 1st, 1989, and the median follow-up time was 7.3 years (maximum 18.3 years). No patient was lost to follow-up.

### *Statistics*

Analyses were performed by various programmes in the statistical package BMDP (Dixon, 1985). Associations between different variables were assessed by Pearson's chi-square test. Survival analysis (life-table method) was done by BMDP-1L using the Mantel-Cox test for differences between groups, and plots for cumulative proportion surviving are given. *Recurrence free survival*, i.e. the time from diagnosis until the appearance of loco-regional recurrences or distant spread, and the *patient survival* (survival time until thyroid cancer deaths) were studied. Deaths from intercurrent disease, without tumour recurrence, were censored in the analysis of recurrence-free survival. The influence of covariates on survival was analysed by the proportional hazards method (Cox & Oakes, 1984) with BMDP-2L, using a forward stepwise procedure. In these analyses, all variables with a $P$-value of 0.10 or less in the life-table studies were included. Estimated regression coefficients and $P$-values are given in the Tables.

### Results

Table I shows the distribution of the cases according to major clinicopathologic variables. Definite extra-thyroidal invasion was present in 19% of the cases, and 45% had lymph node metastases at the time of diagnosis.

Tumour recurrences during the follow-up period occurred in 27 patients (22%). Of the 25 deaths, 14 were due to thyroid cancer (56%).

EGF-receptor immunostaining was found in 120 of 125 cases (96%), whereas five cases were completely negative. In some cases, a weak positivity was found in follicular cells in adjacent non-neoplastic thyroid tissue. Table II shows that a distinct membraneous EGF-receptor staining (grade 2 and 3) was present in 46% of the cases, and the staining was especially evident on the apical surface of the tumour cells (Figure 1). In 90% of the tumours, a cytoplasmic pattern of positive staining was found (Figure 1). This was a diffuse or finely granular staining in most cases, but in some tumours coarse granules were present in the supranuclear part of the cytoplasm. Of the cases with cytoplasmatic positivity, 12% showed a marked (grade 3) staining reaction. Mixed staining patterns were also observed (Figure 1).

Table III shows that the degree of cytoplasmic immunopositivity was significantly associated with the extent of primary tumour infiltration ($P = 0.009$). The frequency of cases with tumour growth in the thyroid capsule or extra-thyroidal invasion was 17%, 63% and 71% in grade 1, 2 and 3 tumours, respectively. Membraneous staining was not associated with any of the variables.

Univariate analysis of recurrence free survival (life-table method) showed that primary tumour extent and lymph node metastases were significant variables (Table IV). Cytoplasmic EGF-receptor staining was also found to be significant, $P = 0.006$ using the Mantel Cox' trend test (Table IV, Figure 2). Grade 3 cytoplasmic staining was significantly different from Grade 2 positivity ($P = 0.05$). Patient survival was associated with sex, age and primary tumour extension, but EGF-receptor staining was not significant (Table IV).

Multivariate survival analysis (Cox' method) of recurrence free survival including sex, age, primary tumour extent, lymph node metastases and cytoplasmic EGF-receptor immunostaining showed that only lymph node metastases ($P = 0.0009$) and EGF-receptor positivity ($P = 0.0048$) remained as significant and independent variables (Table V). EGF-receptor was found to be the strongest prognostic factor, with a regression coefficient of 1.28, compared to 0.81 for lymph node metastases. Multivariate analysis (Cox' method) of patient survival was not performed since EGF-receptor immunostaining turned out to be *not* significant in the univariate life-table study.

**Table I** Distribution of patients with papillary thyroid carcinoma according to important clinicopathologic variables ($n$ = 125)

| *Variable* | n | % |
|---|---|---|
| *Sex* | | |
| males | 36 | 28.8 |
| females | 89 | 71.2 |
| *Age* | | |
| 0–49 years | 71 | 56.8 |
| 50 + years | 54 | 43.2 |
| *Primary tumour extent* | | |
| intra-thyroidal tumour | 51 | 40.8 |
| thyroid capsular invasion | 50 | 40.0 |
| extra-thyroidal invasion | 24 | 19.2 |
| *Lymph node metastases* | | |
| absent | 67 | 54.5 |
| intra-nodal growth | 20 | 16.3 |
| extra-nodal growth | 36 | 29.2 |

### Discussion

The present study of papillary thyroid carcinoma indicates that EGF-receptors are present in a majority of the cases. Corresponding results have been reported by others using biochemical (Duh *et al.*, 1985; Makinen *et al.*, 1988; Masuda *et al.*, 1988; Di Carlo *et al.*, 1990) or immunohistochemical methods (Lemoine *et al.*, 1991; Song *et al.*, 1991; Mizukami *et al.*, 1992). Our results add further evidence to the role of oncogenes in development and progression of thyroid tumours. In a recent study, the presence of transforming growth factor α (TGF-α), a known EGF-R ligand, as well as TGF-α mRNA was found in the epithelial component of papillary carcinomas, indicating an autocrine growth factor production (Haugen *et al.*, in press). In addition to altera-

**Figure 1** Patterns of EGF-receptor immunostaining in papillary thyroid carcinomas. **a**, membraneous staining, × 428; **b**, mixed membraneous and cytoplasmic staining, × 428; **c**, strong (grade 3) cytoplasmic staining, × 428.

tions in the EGF-R system, we have previously shown that c-*erb*B-2 expression is also increased (Haugen *et al.*, 1992).

The pattern and intensity of EGF-receptor immunostaining varied considerably between different tumours. Increased expression in the tumour cell cytoplasm was significantly associated wtih extra-thyroidal growth of the primary tumours. However, no relationship to co-existing lymph node metastases was found, in line with studies on breast cancer (Bolla *et al.*, 1992). Previous reports on carcinomas of the urinary bladder, stomach and large bowel have also suggested an association between EGF-R positivity and invasive growth (Neal *et al.*, 1985; Yasui *et al.*, 1988; Smith *et al.*, 1989; Yonemura *et al.*, 1991), but the mechanism is not clear. Some *in vitro* studies indicate that EGF may stimulate the secretion of proteolytic enzymes (Lee & Weinstein, 1978; Boyd, 1989), but in a study of EGF-R and cathepsin D in endometrial and cervical tumours, no significant relationship was found (Scambia *et al.*, 1991). In addition, cellular migration may be stimulated by EGF (Westermark *et al.*, 1982).

Positivity for EGF-receptor was significantly related to increased risk of recurrent disease in papillary thyroid carcinomas, in contrast to a recent study where no prognostic importance could be found (Mizukami *et al.*, 1992). Our present results are thus in general agreement with reports on breast cancer, where the presence of EGF-receptors has been documented to be an important predictor of tumour recurrences as well as patient survival (Sainsbury *et al.*, 1987; Grimaux *et al.*, 1989; Nicholson *et al.*, 1991; Toi *et al.*, 1991). Further studies using quantitative methods should now be performed.

The mechanism of EGF-receptor influence on patient prognosis has not yet been clarified. In breast cancer, increased expression of EGF-R has been related to dedifferentiation of tumour cells, with increased cellular atypia,

**Table II** Patterns and intensity of EGF-receptor immunostaining in papillary thyroid carcinoma ($n$ = 125)

| | *Membraneous staining* | | *Cytoplasmic staining* | |
|---|---|---|---|---|
| *Staining*[a] | n | % | n | % |
| Grade 1 | 67 | 53.6 | 12 | 9.6 |
| Grade 2 | 46 | 36.8 | 99 | 79.2 |
| Grade 3 | 12 | 9.6 | 14 | 11.2 |

[a]Grade 1 = no staining; Grade 2 = slight or moderate staining; Grade 3 = marked staining.

**Table III** Associations between pattern of EGF-receptor immunostaining and important clinicopathologic variables in patients with papillary thyroid carcinoma ($n$ = 125)

| | P-*value*[a] | |
|---|---|---|
| *Variables* | *Membraneous staining*[b] | *Cytoplasmic staining*[b] |
| Sex | 0.9 | 0.8 |
| Age[c] | 0.11 | 0.9 |
| Primary tumour extent[d] | 0.2 | 0.009[e] |
| Lymph node metastases[f] | 0.5 | 0.4 |

[a]Pearson's chi-square. [b]Grades 1, 2, 3 (see Material and methods). [c]0–49 years, 50 + years. [d]intra-thyroidal, thyroid capsular invasion, extra-thyroidal invasion. [e]For details, see Results. [f]absent, intra-nodal growth, extra-nodal growth.

**Figure 2** Recurrence-free survival according to cytoplasmic EGF-receptor immunostaining in papillary thyroid carcinomas.

**Table IV** Univariate survival analysis (life-table method) of patients with papillary thyroid carcinoma according to clinicopathologic variables and EGF-receptor immunostaining, $n$ = 125 (the figures in parenthesis give the number of patients who were alive after 10 years and eligible for estimation of 10-year survival; the *P*-values correspond with a standard life-table analysis, based on all patients and observed events during the whole follow-up period)

| *Variables* | n | *10-year recurrence free survival (%)*[a] | P[b] | *10-year patient survival (%)* | P[b] |
|---|---|---|---|---|---|
| *Sex* | | | 0.2 | | 0.014 |
| females | 89 (28) | 76.5 | | 91.6 | |
| males | 36 (10) | 66.0 | | 81.6 | |
| *Age* | | | 0.05 | | <0.00005 |
| 0–49 years | 71 (25) | 81.4 | | 98.2 | |
| 50 + years | 54 (13) | 61.7 | | 75.8 | |
| *Primary tumour extent* | | | 0.0001 | | 0.010 |
| intra-thyroidal | 51 (22) | 95.5 | | 98.0 | |
| thyroid capsule invasion | 50 (12) | 50.3 | | 81.7 | |
| extra-thyroidal invasion | 24 (4) | 65.3 | | 80.9 | |
| *Lymph node metastases* | | | <0.00005 | | 0.3 |
| absent | 67 (22) | 88.8 | | 93.3 | |
| intra-nodal growth | 20 (6) | 82.2 | | 79.8 | |
| extra-nodal growth | 36 (9) | 33.3 | | 84.0 | |
| *EGF-receptor immunostaining*[c] | | | | | |
| membraneous staining | | | 0.7 | | 0.3 |
| grade 1 | 67 (16) | 73.9 | | 83.5 | |
| grade 2 | 46 (17) | 76.6 | | 92.8 | |
| grade 3 | 12 (5) | 63.5 | | 100.0 | |
| cytoplasmic staining | | | 0.006 | | 0.11 |
| grade 1 | 12 (7) | 100.0 | | 100.0 | |
| grade 2 | 99 (25) | 73.5 | | 85.5 | |
| grade 3 | 14 (6) | 47.0 | | 100.0 | |

[a]Of 125 cases included, nine were not radically treated and excluded in the analyses of recurrence free survival. [b]Mantel-Cox' test. [c]Trend version of the Mantel-Cox' test.

**Table V** Multivariate analysis of recurrence free survival (Cox' proportional hazards method) of patients with papillary thyroid carcinoma ($n = 113$)[a]

| *Variables* | *Regression coefficient* | *Standard error* | P-*value*[b] |
|---|---|---|---|
| Lymph node metastases[c] | 0.81 | 0.26 | 0.0009 |
| EGF-receptor[d] (cytoplasmic staining) | 1.28 | 0.43 | 0.0048 |

[a]Of 125 cases, nine were not radically treated and were therefore excluded, and three cases were excluded due to lack of information. [b]L ratio test of significance. [c]Categories: absent, intra-nodal growth, extra-nodal growth. [d]Categories: grade 1, grade 2, grade 3.

reduced concentration of oestrogen receptors and a higher proliferative fraction as measured by Ki-67 immunopositivity (Fitzpatrick *et al.*, 1984; Bolla *et al.*, 1992). An inverse correlation between EGF-R content and TSH-response has also been noted in anaplastic thyroid carcinomas (Di Carlo *et al.*, 1990). These findings indicate that increased EGF-R expression is associated with proliferative activity in tumour cells and reduced dependency of normal growth regulators.

Interestingly, two specific staining patterns were observed in the present study. A distinct membraneous positivity was found in 46% of the cases, whereas a diffuse or granular cytoplasmic staining was observed in 90% of all tumours. These patterns have been briefly noted by others (Kashima *et al.*, 1991; Mizukami *et al.*, 1992). Only the cytoplasmatic form of staining, however, showed a significant association with patient prognosis. Earlier studies (Lemoine *et al.*, 1990; Aasland *et al.*, 1988; Aasland *et al.*, 1990) indicate that increased expression of EGF-R in thyroid tumours is not due to gene amplification or gross rearrangements. Therefore, epigenetic changes may be involved, and recent Western blot studies of fresh material indicate that modified proteins are present in some cases (Haugen *et al.*, in preparation).

In conclusion, the present study using semiquantitative immunohistochemical assessment indicates that expression of EGF-receptors may be an important feature of papillary thyroid carcinomas, and various staining patterns seem to be of different biological significance. Strong cytoplasmatic immunopositivity was associated with extra-thyroidal tumour invasion and found by multivariate analysis to be the strongest independent predictor of recurrent disease. However, quantitative methods should be used in further studies to establish this association.

This study has been supported by the *Norwegian Cancer Society*. We thank Tor Christensen, Inger Hernar, Anne-Marie Larsen and Bendik Nordanger for excellent technical assistance.

### References

AASLAND, R., LILLEHAUG, J.R., MALE, R., JØSENDAL, O., VARHAUG, J.E. & KLEPPE, K. (1988). Expression of oncogenes in thyroid tumours: co-expression of c-*erb*B-2/*neu* and c-*erb*B. *Br. J. Cancer*, **57**, 358-363.

AASLAND, R., AKSLEN, L.A., VARHAUG, J.E. & LILLEHAUG, J.R. (1990). Co-expression of the genes encoding transforming growth factor-alpha and its receptor in papillary carcinomas of the thyroid. *Int. J. Cancer*, **46**, 382-387.

AKSLEN, L.A., HALDORSEN, T., THORESEN, S.Ø. & GLATTRE, E. (1991). Survival and causes of death in thyroid cancer: a population-based study of 2479 cases from Norway. *Cancer Res.*, **51**, 1234-1241.

AKSLEN, L.A., MYKING, A.O., SALVESEN, H. & VARHAUG, J.E. (1993). Prognostic importance of various clinicopathological features in papillary thyroid carcinoma. *Eur. J. Cancer*, **29A**, 44-51.

BOLLA, M., CHEDIN, M., COLONNA, M., MARRON, J., ROSTAING-PUISSANT, B. & CHAMBAZ, E. (1992). Prognostic value of epidermal growth factor receptor in a series of 303 breast cancers. *Eur. J. Cancer*, **28**, 1052-1054.

BOYD, D. (1989). Examination of the effects of epidermal growth factor on the production of urokinase and the expression of the plasminogen activator receptor in a human colon cancer cell line. *Cancer Res.*, **49**, 2427-2432.

BYAR, D.P., GREEN, S.B., DOR, P., WILLIAMS, E.D., COLON, J., VAN GILSE, H., MAYER, A., SYLVESTER, M. & GLABBEKE, M.V. (1979). A prognostic index for thyroid carcinoma. A study of the E.O.R.T.C. thyroid cancer cooperative group. *Eur. J. Cancer*, **15**, 1033-1041.

CARCANGIU, M.L., ZAMPI, G., PUPI, A., CASTAGNOLI, A. & ROSAI, J. (1985). Papillary carcinoma of the thyroid. A clinicopathologic study of 241 cases treated at the University of Florence, Italy. *Cancer*, **55**, 805-828.

CARPENTER, G. & COHEN, S. (1979). Epidermal growth factor. *Ann. Rev. Biochem.*, **48**, 193-216.

COX, D.R. & OAKES, D. (1984). *Analysis of Survival Data*. Chapman and Hall: London.

DI CARLO, A., MARIANO, A., PISANO, G., PARMEGGIANI, U., BEGUINOT, L. & MACCHIA, V. (1990). Epidermal growth factor receptor and thyrotropin response in human thyroid tissues. *J. Endocrinol. Invest.*, **13**, 293-299.

DIXON, W.J. (1985) (chief ed.). *BMDP Statistical Software*. University of California Press.

DUH, Q.-Y., GUM, E.T., GEREND, P.I., RAPER, S.E. & CLARK, O.H. (1985). Epidermal growth factor receptors in normal and neoplastic thyroid tissue. *Surgery*, **98**, 1000-1007.

FITZPATRICK, S.L., BRIGHYWELL, J., WITTLIFF, J.L., BARROWS, G.M. & SCHULTZ, G.S. (1984). Epidermal growth factor binding by breast tumor biopsies and relationship to estrogen receptor and progestin receptor. *Cancer Res.*, **44**, 3448.

GRIMAUX, M., ROMAIN, S., REMVIKOS, Y., MARTIN, P.M. & MAGDELENAT, H. (1989). Prognostic value of epidermal growth factor receptor in node positive breast cancer. *Br. Cancer Res. Treat.*, **14**, 77-90.

HAUGEN, D.F., AKSLEN, L.A., VARHAUG, J.E. & LILLEHAUG, J. (1992). Expression of c-*erb*B-2 protein in papillary thyroid carcinomas. *Br. J. Cancer*, **65**, 832-837.

HAUGEN, D.F., AKSLEN, L.A., VARHAUG, J.E. & LILLEHAUG, J. (1993). Demonstration of a TGF-α – EGF receptor autocrine loop and c-*myc* protein overexpression in papillary thyroid carcinomas. *Int. J. Cancer* (in press).

HEDINGER, C. (1988). (ed). *Histological Typing of Thyroid Tumours*, WHO, Springer-Verlag: Berlin, Heidelberg.

KASHIMA, K., YOKOYAMA, S., NAKAYAMA, I. & NOGUCHI, S. (1991). Immunohistochemical study on expression of c-*myc*, p53, c-*erb*B-2 and epidermal growth factor receptor in human thyroid tumors. *Acta Histochem. Cytochem.*, **24**, 563-570.

LEE, L.S. & WEINSTEIN, I.B. (1978). Epidermal growth factor, like phorbol esters, induces plasminogen activator in HeLa cells. *Nature*, **274**, 696-697.

LEMOINE, N.R., WYLLIE, F.S., LILLEHAUG, J.R. & 8 others (1990). Absence of abnormalities of the c-*erb*B-1 and c-*erb*B-2 proto-oncogenes in human thyroid neoplasia. *Eur. J. Cancer*, **26**, 777-779.

LEMOINE, N.R., HUGHES, C.M., GULLICK, W.J., BROWN, C.L. & WYNFORD-THOMAS, D. (1991). Abnormalities of the EGF receptor system in human thyroid neoplasia. *Int. J. Cancer*, **49**, 558-561.

MAKINEN, T., PEKONEN, F., FRANSSILA, K. & LAMBERG, B.A. (1988). Receptors for epidermal growth factor and thyrotropin in thyroid carcinoma. *Acta Endocrinol.*, **117**, 45-50.

MASUDA, H., SUGENOYA, A., KOBAYASHI, S., KASUGA, Y. & IIDA, F. (1988). Epidermal growth factor receptor on human thyroid neoplasms. *World J. Surg.*, **12**, 616-622.

MIZUKAMI, Y., NONOMURA, A., HASHIMOTO, T., MICHIGISHI, T., NOGUCHI, M., MATSUBARA, F. & YANAIHARA, N. (1991). Immunohistochemical demonstration of epidermal growth factor and c-myc oncogene product in normal, benign and malignant thyroid tissues. *Histopathology*, **18**, 11-18.

MIZUKAMI, Y., NONOMURA, A., MICHIGISHI, T., YOKOYAMA, K., NOGUCHI, M., HASHIMOTO, T., NAKAMURA, S. & MATSUBARA, F. (1992). Immunohistochemical demonstration of epidermal growth factor receptors in normal, benign and malignant thyroid tissues. *Int. J. Oncol.*, **1**, 331-335.

NEAL, D.E., MARSH, C., BENNET, M.K., ABELL, P.D., HALL, R.R. & SAINSBURY, J.R.C. (1985). Epidermal growth factor receptors in human bladder cancer: comparison of invasiveness and superficial tumors. *Lancet*, **i**, 366-368.

NICHOLSON, S., RICHARD, J., SAINSBURY, C. & 7 others (1991). Epidermal growth factor receptor (EGFR) results of a 6 year follow-up study in operable breast cancer with emphasis on the node negative subgroup. *Br. J. Cancer*, **63**, 146–150.

SAINSBURY, J.R.C., FARNDON, J.R., NEEDHAM, G.K., MALCOLM, A.J. & HARRIS, A.L. (1987). Epidermal growth factor receptor status as predictor of early recurrence of and death from breast cancer. *Lancet*, **i**, 1398–1402.

SCAMBIA, G., PANICI, P.B., FERRANDINA, G., BAIOCCHI, G., DISTEFANO, M. & MANCUSO, S. (1991). Cathepsin D in primary endometrial and cervical tumors: relationship with histopathological parameters and with estrogen, progesterone, and epidermal growth factor receptor. *Cancer J.*, **4**, 178–182.

SCHINDLER, A.-M., VANMELLE, G., EVEQUOZ, B. & SCAZZIGA, B. (1991). Prognostic factors in papillary carcinoma of the thyroid. *Cancer*, **68**, 324–330.

SMITH, K., FENNELLY, J.A., NEAL, D.E., HALL, R.R. & HARRIS, A.L. (1989). Characterization and quantitation of the epidermal growth factor receptor in invasive and superficial bladder tumors. *Cancer Res.*, **49**, 5810–5815.

SONG, B. (1991). Immunohistochemical demonstration of epidermal growth factor receptor and ceruloplasmin in thyroid diseases. *Acta Pathol. Jpn.*, **41**, 336–343.

TENNVALL, J., BIØRKLUND, A., MØLLER, T., RANSTAM, J. & ÅKERMAN, M. (1985). Prognostic factors of papillary, follicular and medullary carcinomas of the thyroid gland. Retrospective multivariate analysis of 216 patients with a median follow-up of 11 years. *Acta Radiol. Oncol.*, **24**, 17–24.

TOI, M., OSAKI, A., YAMADA, H. & TOGE, T. (1991). Epidermal growth factor receptor expression as a prognostic indicator in breast cancer. *Eur. J. Cancer*, **27**, 977–980.

TUBIANA, M., SCHLUMBERGER, M., ROUGIER, P., LAPLANCHE, A., BENHAMOU, E., GARDET, P., CAILLOU, B., TRAVAGLI, J. & PARMENTIER, C. (1985). Long-term results and prognostic factors in patients with differentiated thyroid carcinoma. *Cancer*, **55**, 794–804.

WESTERMARK, B., MAGNUSSON, A. & HELDIN, C.-H. (1982). Effect of epidermal growth factor on membrane motility and cell locomotion in cultures of human clonal glioma cells. *J. Neurosci. Res.*, **8**, 491–507.

WYNFORD-THOMAS, D. (1991). Oncogenes and anti-oncogenes: the molecular basis of tumour behaviour. *J. Pathol.*, **165**, 187–201.

YASUI, W., SUMIYOSHI, H., HATA, J., KAMEDA, T., OCHIAI, A., ITO, H. & TAHARA, E. (1988). Expression of epidermal growth factor receptor in human gastric and colonic carcinomas. *Cancer Res.*, **48**, 137.

YONEMURA, Y., SUGIYAMA, K., FUSHIDA, S., KAMATA, T., OHOYAMA, S., KIMURA, H., YAMAGUCHI, A. & MIYAZAKI, I. (1991). Tissue status of epidermal growth factor and its receptor as an indicator of poor prognosis in patients with gastric cancer. *Anal. Cell. Pathol.*, **3**, 343–350.